# MoS_2_-PtX_2_ Vertical Heterostructures

**DOI:** 10.3390/nano15181415

**Published:** 2025-09-15

**Authors:** Nikolay Minev, Blagovest Napoleonov, Dimitre Dimitrov, Vladimira Videva, Velichka Strijkova, Denitsa Nicheva, Ivalina Avramova, Tamara Petkova, Vera Marinova

**Affiliations:** 1 Institute of Optical Materials and Technologies, Bulgarian Academy of Sciences, 1113 Sofia, Bulgariabnapoleonov@iomt.bas.bg (B.N.); vvideva@iomt.bas.bg (V.V.); vily@iomt.bas.bg (V.S.); vmarinova@iomt.bas.bg (V.M.); 2 Institute of Solid State Physics, Bulgarian Academy of Sciences, 1784 Sofia, Bulgaria; 3 National Centre of Excellence Mechatronics and Clean Technologies, 8 Kliment Ohridski Blvd., Blk. 8, 1756 Sofia, Bulgaria; 4 Faculty of Chemistry and Pharmacy, Sofia University “St. Kliment Ohridski”, 1164 Sofia, Bulgaria; 5 Institute of Electrochemistry and Energy Systems, Bulgarian Academy of Sciences, 1113 Sofia, Bulgaria; d.vladimirova@iees.bas.bg (D.N.); tpetkova@iees.bas.bg (T.P.); 6 Institute of General and Inorganic Chemistry, Bulgarian Academy of Sciences11, Acad. G. Bonchev Str., 1113 Sofia, Bulgaria; iva@svr.igic.bas.bg

**Keywords:** 2D heterostructure, PtSe_2_, PtTe_2_, MoS_2_, Raman spectroscopy, XPS

## Abstract

This study reports the successful fabrication and characterization of two-dimensional (2D) vertical heterostructures composed of a semiconducting molybdenum disulfide (MoS_2_) layer stacked with semimetallic platinum dichalcogenides (PtSe_2_ and PtTe_2_). The heterostructures were created using a versatile fabrication method that combines chemical vapor deposition (CVD) to grow high-quality MoS_2_ nanolayers with thermally assisted conversion (TAC) for the synthesis of the Pt-based layers. The final MoS_2_/PtSe_2_ and MoS_2_/PtTe_2_ heterostructures were then assembled via a dry transfer process, ensuring high structural integrity. The quality and properties of these heterostructures were investigated using a range of advanced spectroscopic techniques. Raman spectroscopy confirmed the presence of characteristic vibrational modes for each material, validating successful formation. X-ray photoelectron spectroscopy (XPS) analysis further confirmed the elemental composition and oxidation states, though it also revealed the presence of elemental Pt^0^ and oxidized Te^+4^ in the PtTe_2_ layer, suggesting an incomplete conversion. Importantly, the photoluminescence (PL) spectra showed a significant quenching effect, a clear sign of strong interlayer charge transfer, which is essential for optoelectronic applications. Finally, UV-Vis-NIR spectrophotometry demonstrated the combined optical properties of the stacked layers, with the Pt-based layers causing broadening and a blue-shift in the MoS_2_ exciton peaks, indicating altered electronic and optical behavior. This research provides valuable insights into the synthesis and fundamental properties of MoS_2_/PtX_2_ heterostructures, highlighting their potential for next-generation electronic and optoelectronic devices.

## 1. Introduction

The surge of interest in two-dimensional (2D) materials has led to significant breakthroughs across various research fields. Among these, transition metal dichalcogenides (TMDs) have emerged as a highly versatile and promising class due to their unique electronic, optical, mechanical, and catalytic properties [1,2,3]. Composed of a transition metal atom (M) sandwiched between two chalcogenide atoms (X), with the general formula MX_2_, TMDs exhibit a wide range of behaviors—from semiconducting and semimetallic to superconducting—dependent on their composition and structural phase. These atomically thin layers, often just a few atoms thick, display properties distinctly different from their bulk counterparts. Notably, the properties of 2D materials can be tailored to specific applications through careful modification of their surface chemistry and structure [4,5,6,7].

Recently, synthesis technologies and efficient growth strategies for wafer-scale TMDs and heterojunctions have been comprehensively summarized and analyzed. These are particularly important for device research and development [8]. However, further exploration and research are still needed to integrate two-dimensional (2D) materials into practical semiconductor applications. This includes maintaining high carrier mobility with wafer-scale uniformity and achieving high-quality, low-defect, low-temperature growth. Leveraging the weak van der Waals forces that hold different TMD layers together, researchers have pioneered the fabrication of innovative heterostructures through vertical stacking or lateral assembly of diverse monolayers/few layers of 2D materials [9,10]. These engineered structures further enable precise control over electronic band alignments, light–matter interactions, and charge transfer processes, offering the pathways to realize advanced functionalities [11,12]. The interlayer van der Waals bonding of 2D materials enables the construction of heterostructures without consideration of lattice mismatch [13,14], which provides a suitable strategy for heterostructures that combine the advantages of different 2D materials. In order to circumvent some weaknesses of single 2D materials, it is promising to construct 2D van der Waals heterostructures with different types of materials [15,16]. As a result, TMD heterostructures have become a vibrant frontier in materials science, with significant potential for applications in electronics, optoelectronics, energy harvesting, catalysis, and sensing. Specifically, semimetal/semiconductor 2D heterostructures are widely used in the design of electronic and optoelectronic devices to achieve high-sensitivity photodetection [17]. Molybdenum disulfide (MoS_2_), the most studied among 2D chalcogenide compounds, presents outstanding electrical and optical properties. It has been stacked with graphene, SnS_2_, TiS_3_, CdS, and so on to fabricate heterojunctions [18,19]. In addition to MoS_2_, platinum diselenide (PtSe_2_) is an important member of group-10 TMDs. The bandgaps of monolayer PtSe_2_ and bilayer PtSe_2_ are 1.2 eV and 0.21 eV, respectively. Moreover, when the number of layers reaches three and above, it exhibits semimetal characteristics [18], which enables sensitivity to the near-infrared (NIR) region. PtSe_2_ is a p-type semiconductor characterized by high carrier mobility (>1000 cm^2^ V^−1^ s ^−1^), a narrow bandgap (0.2 eV in multilayer PtSe_2_), and strong light-absorption properties at room temperature [19], which has the potential to functionalize as the photoactive layer in a p-n heterojunction with MoS_2_.

As-grown MoS_2_ is an n-type semiconductor, and PtSe_2_ is a p-type semiconductor characterized by high carrier mobility. Platinum ditelluride (PtTe_2_) can be either p-type or n-type, depending on the specific growth conditions and the presence of defects. It is often considered a p-type material due to the presence of intrinsic defects that lead to hole doping. However, PtTe_2_ can also be tuned to become n-type, for example, by using it as a contact in devices like atomic transistors [20,21,22].

Semiconducting TMDCs with group VIB transition metals (M = Mo, W) have been extensively explored. Unfortunately, the experimental carrier mobility obtained from the related devices is always one order of magnitude lower than the theoretical value [23], which seriously hinders further device improvement. In addition, the energy bandgap of MoS_2_, WS_2,_ etc., only covers the visible to near-infrared spectrum (<1.0 μm), which limits their applications in the longer wavelength. Recently, the newly discovered group-10 TMDCs with strong interlayer interaction, tunable bandgap from 0.0 to 1.60 eV, and high carrier mobility at room temperature have aroused increasing research attention [24].

This article explores the preparation of MoS_2_ and Pt-based group 10 TMDs (PtSe_2_, PtTe_2_), and their heterostructures synthesized by using the CVD (chemical vapor deposition) technique combined with the transfer technology. The assembled vdW heterostructures of MoS_2_-PtSe_2_ and MoS_2_-PtTe_2_ were thoroughly characterized to assess heterostructure quality and possible applications.

## 2. Materials and Methods

**Synthesis of MoS_2_ via Chemical Vapor Deposition (CVD)**

The synthesis of MoS_2_ nanofilms [5] was conducted in a precisely controlled two-zone chemical vapor deposition (CVD) system to ensure high-quality crystalline growth with minimal defects. The process incorporated perylene-3,4,9,10-tetracarboxylic acid tetrapotassium salt (PTAS–C_24_H_8_K_4_O_8_) as a seeding promoter, spin-coated onto the substrate to enhance nucleation efficiency and promote lateral growth. Zone 2 was initially heated to 105 °C for 60 min under a 300 sccm argon purge to remove contaminants. Then, the temperature was increased to 770 °C over 45 min while reducing argon flow to 25 sccm, optimizing conditions for MoS_2_ nucleation. In parallel, Zone 1, containing sulfur, was heated to 180 °C, starting 10 min before Zone 2 reached its final temperature, to ensure sufficient sulfur vapor for the precursor reaction.

Upon reaching the target conditions, Zone 2 was held at 770 °C for 5 min under a constant argon flow of 25 sccm, ensuring complete precursor reaction and uniform MoS_2_ layer formation. Precise control of temperature, precursor flux, and gas-phase dynamics is critical for optimizing crystallographic quality and film uniformity.

**Synthesis of PtSe_2_ and PtTe_2_ nanofilms/flakes**

PtSe_2_ and PtTe_2_ thin films were synthesized via thermally assisted conversion (TAC) of pre-deposited Pt films in a three-zone gradient tube furnace [6,7]. High-purity selenium (99.999%) and tellurium (99.999%) served as chalcogen sources. The Pt-coated substrates were placed in the central zone at 500 °C, while the chalcogenide sources were positioned upstream at controlled temperatures to regulate vapor pressure. The reaction was conducted over 2 h, with optimized heating and cooling rates. A carrier gas mixture of Ar (90%) and H_2_ (10%) at 150 sccm facilitated chalcogen incorporation while suppressing oxidation. The reactor was purged before initiation to remove residual contaminants, ensuring a clean growth environment and preventing unwanted secondary reactions.

**Transfer of MoS_2_ Thin Films**

MoS_2_ thin films were transferred from sapphire substrates onto Pt-based group 10 transition metal dichalcogenide layers (PtSe_2_, PtTe_2_) to form heterostructures using the well-known thermal release tape (TRT) transfer process.

**Figure 1:**
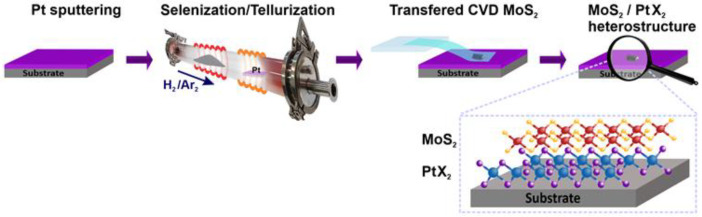
CVD setup and heterostructure assembling method.

The resulting MoS_2_-based heterostructures (see Figure 1) exhibited high structural integrity and minimal contamination, making them suitable for Raman spectroscopy, photoluminescence analysis, and electronic transport measurements.

Atomic force microscopy (AFM) with an MFP-3D system (Asylum Research, Oxford Instruments, Santa Barbara, CA, USA) was employed to investigate the surface topography and thickness of the deposited layers.

Raman spectral data were collected using a laser confocal microscopic Raman spectrometer (inVia Qontor) (Renishaw Wotton-under-Edge, UK) The 532 nm, 50 mW laser was used for excitation. This device included a high-speed grating feedback platform (MS-30), a high-resolution microscopic system (Leica, Wetzlar, Germany), and a CCD detector (Renishaw, RenCam series, Wotton-under-Edge, UK). During the experiment, the laser beam was focused on the sample surface with a 100× objective lens, exciting the sample and producing Raman scattering signals. All Raman spectra in this study were recorded in a temperature-controlled dark room with the following parameters: a 2400 L/mm high-resolution grating and an exposure time of 10 s per spectrum.

The X-ray photoelectron spectroscopy (XPS) studies were performed in a VG Escalab MKII electron spectrometer (Thermo Fisher Scientific Inc., Waltham, MA, USA) using AlKα radiation with an energy of 1486.6 eV under a base pressure of 10^−8^ Pa and a total instrumental resolution of 1 eV. The binding energies (BEs) were determined using the O1s line as a reference with an energy level of 530.3 eV. The accuracy of the measured BE was 0.2 eV. The photoelectron lines of the constituent elements on the surface were recorded and corrected by subtracting a Shirley-type background and quantified using the peak area and Scofield’s photoionization cross-sections. The deconvolution of spectra was performed with XPSPEAK41 software.

The optical transmittance spectra were measured at room temperature using an Ultraviolet–Visible–Near-infrared (UV-VIS-NIR) spectrophotometer Cary 5E (Agilent Technologies, Inc, Santa Clara, CA, USA) in the wavelength range of 200 nm–800 nm.

## 3. Results

### 3.1. AFM Analysis

**Figure 2:**
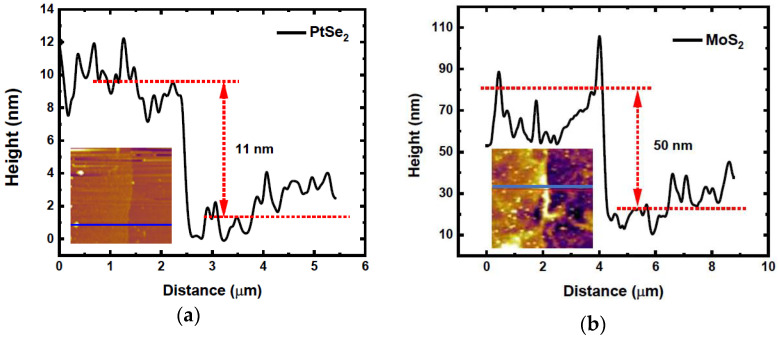
Height profiles of (**a**) PtSe_2_ and (**b**) MoS_2_. Insets: related 2D AFM images.

**Figure 3:**
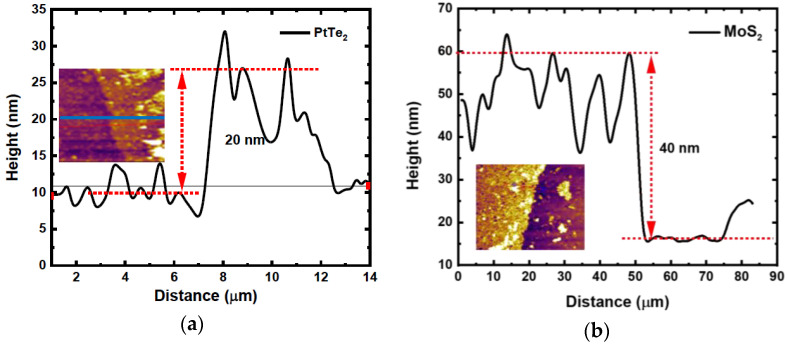
Height profiles of (**a**) PtTe_2_ and (**b**) MoS_2_. Insets: related 2D AFM images.

The surface topography was obtained by AFM. Figure 2 and Figure 3 clearly show the thin layers of MoS_2_, PtSe_2_, and PtTe_2_ flakes. The obtained thickness estimated by height profiles is indicated in the figures. From these measurements, the combined height of the heterostructures is around ~60 nm.

### 3.2. Raman Spectroscopy

**Figure 4:**
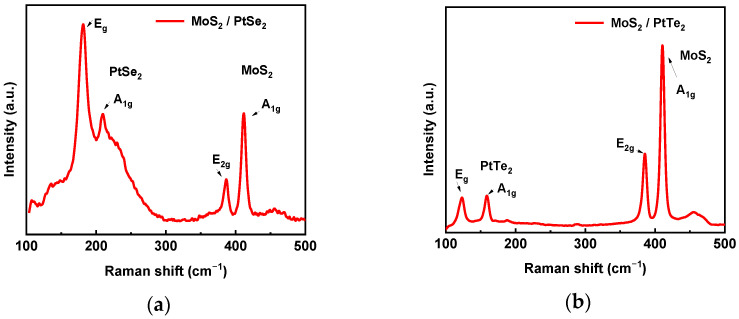
(**a**) Raman spectrum of MoS_2_/PtSe_2_ heterostructure at room temperature using a green laser source (532 nm). Characteristic peaks of MoS_2_ (E_2g_ at 387 cm^−1^ and A_1g_ at 412 cm^−1^) and PtSe_2_ (E_g_ at 177 cm^−1^ and A_1g_ at 206 cm^−1^). (**b**). Raman spectrum of MoS_2_/PtTe_2_ heterostructure at room temperature using a green laser source (532 nm). Characteristic peaks of MoS_2_ (E_2g_ at 386 cm^−1^ and A_1g_ at 410 cm^−1^) and PtTe_2_ (E_g_ at 123 cm^−1^ and A_1g_ at 159 cm^−1^).

Raman spectroscopy was carried out on both MoS_2_/PtSe_2_ and MoS_2_/PtTe_2_ heterostructures to confirm the successful formation and quality after the fabrication. The Raman spectrum of the MoS_2_/PtSe_2_ heterostructure in Figure 4a shows vibrational peaks from both PtSe_2_ and MoS_2_. The spectra of PtSe_2_ films exhibit two characteristic peaks at approximately 177 cm^−1^ and 206 cm^−1^, which represent the E_g_ and A_1g_ modes of layered PtSe_2_, respectively [19]. The E_g_ peak originates from the in-plane vibration of selenium (Se) atoms, and the A_1g_ peak is caused by the out-of-plane vibration of Se atoms. A red-shift in the position of both peaks and an increase in the intensity ratio of the two peaks are observed as the number of PtSe_2_ layers increases [18]. This behavior can be explained by an increasing out-of-plane contribution due to an increase in van der Waals interactions between the layers [19]. The full width at half-maximum (FWHM) of the E_g_ peak indicates the material quality of PtSe_2_ [19]. For high-quality TAC-grown PtSe_2_ films, the FWHM is smaller than 5 cm^−1^ [20]. The 387 cm^−1^ (E_2g_) and 412 cm^−1^ (A_1g_) peaks are from MoS_2_, with a spacing of 25 cm^−1^, characteristic of few-layer thickness [22].

Raman spectroscopy was used to further characterize the multilayered MoS_2_/PtTe_2_ heterostructure. As shown in Figure 4b, two distinct Raman peaks located at approximately 386 cm^−1^ and 410 cm^−1^ correspond to E_2g_ and A_1g_ modes for multilayer MoS_2_; the in-plane (E_2g_) mode corresponds to the sulfur atoms vibrating in one direction and the molybdenum atom in the other, while the out-of-plane (A_1g_) mode is a mode of the sulfur atoms vibrating out-of-plane. The two distinct Raman peaks located at ~123 cm^−1^ and ~159 cm^−1^ correspond to E_g_ and A_1g_ modes for PtTe_2_ [23].

### 3.3. Photoluminescence

**Figure 5:**
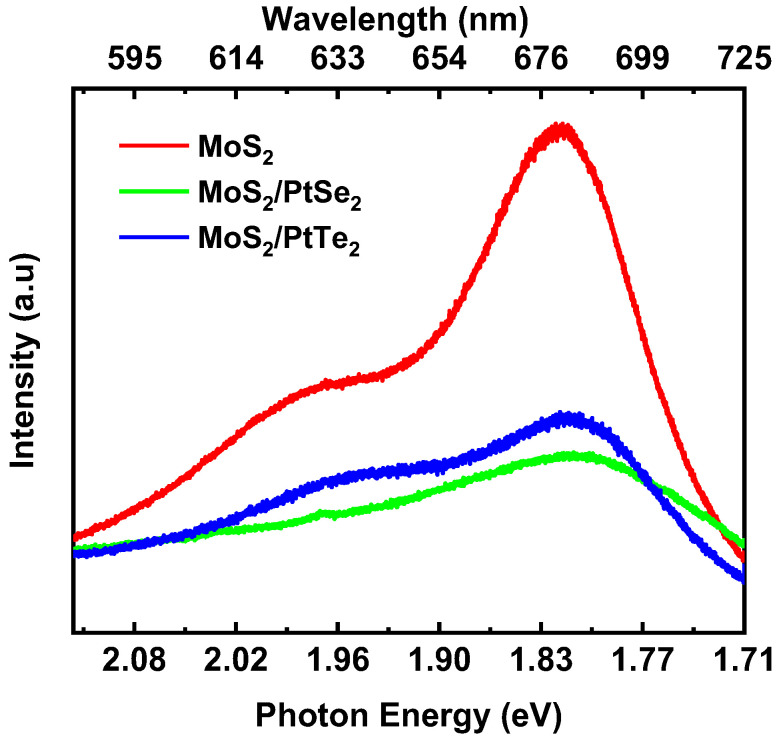
Photoluminescence spectra of MoS_2_ and MoS_2_/PtSe_2_ and MoS_2_/PtTe_2_ heterostructures.

To further investigate the photophysical properties of this heterojunction, the photoluminescence (PL) spectra of MoS_2_/PtSe_2_, and MoS_2_/PtTe_2_ were collected under 532 nm laser illumination, respectively. As shown in Figure 5, the MoS_2_ layer exhibited strong PL emission. These results show that the interlayer interactions in MoS_2_/PtSe_2_ and MoS_2_/PtTe_2_ heterostructures are proved by the PL measurements. PL quenching is a common photophysical phenomenon in van der Waals heterostructures, ascribed to interlayer charge transfer that reduces the radiative recombination efficiency of MoS_2_ [25,26]. These spectroscopic results indicate the successful construction of the MoS_2_/PtSe_2_ and MoS_2_/PtTe_2_ heterostructures with strong interlayer interaction.

### 3.4. XPS Analysis

**Figure 6:**
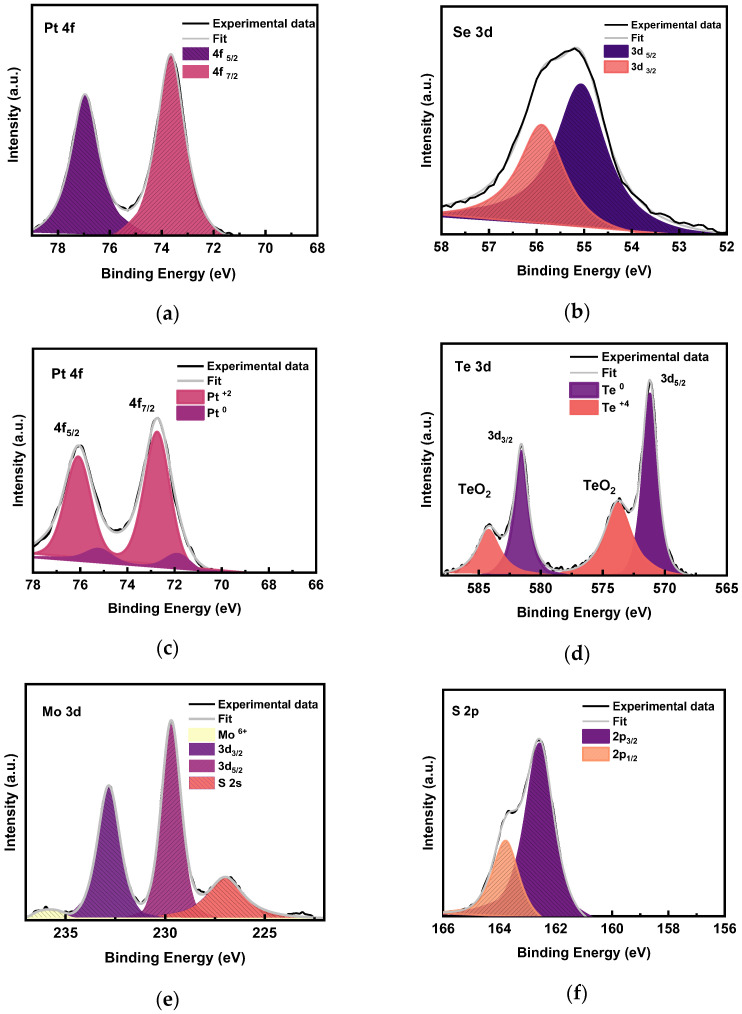
XPS spectra of Pt 4f and Se 3d core levels of PtSe_2_ (**a**,**b**); Pt 4f and Te 3d core levels of PtTe_2_ (**c**,**d**); and Mo 3d and S 2p core levels of MoS_2_ (**e**,**f**).

The XPS analyses of PtSe_2_, PtTe_2_, and MoS_2_ were carried out to determine the identity and oxidation states of the elements. The PtSe_2_ high-resolution spectrum of Pt in Figure 6a shows the presence of two types of platinum oxidation states. The peak pair at ~76.2 eV and 72.9 eV is attributed to Pt^2+^. There were no discernable peaks at 75.2 eV and 71.9 eV attributed to elemental platinum (Pt^0^) [21], signifying a full conversion of the base Pt to PtSe_2_. We can conclude that there is no presence of oxidized Pt within the PtSe_2_ samples due to the absence of the PtO_2_ peaks at ~73.9 eV and ~77.3 eV. The high-resolution scan of Se in Figure 6b shows a broad peak that is deconvoluted into two peaks at 55.1 eV and 55.9 eV. These peaks are consistent with the Se 3d_5/2_ and Se 3d_3/2_ peaks observed for various metal selenide materials [19].

XPS measurements carried out on PtTe_2_ are shown in Figure 6c,d. The Pt high-resolution scan (Figure 6c) displays two dominant peaks at 75.7 eV and 72.5 eV, which are characteristic of Pt^2+^ [19]. The two low-area peak pairs at 74.2 eV and 71.5 eV are attributed to Pt^0^ [20]. These results are consistent with the observations reported in [27]. The XPS high-resolution spectrum of tellurium shown in Figure 6d illustrates the presence of two Te oxidation states in PtTe_2_. The peak at 571 eV and its corresponding shadow peak at 581 eV with a peak-to-peak spacing of 10 eV is due to elemental Te (0 oxidation state) [28]. On the other hand, the peaks at 574 eV and 584 eV occur because of the presence of oxidized tellurium in the prepared material. The oxidation state of Te in its oxidized form is +4 [29,30]. These observations suggest the presence of only Te (0) and Te (+4) in PtTe_2_. Due to the strong covalent nature of the Pt-Te bond, its electronic structure is better described as Pt metal possessing an oxidation state within the range of 0 to +2 rather than +4, while the Te chalcogenide adopts an oxidation state of about 0 instead of 2 [20]. Unreacted tellurium (Te) within a MoS_2_/PtTe_2_ heterostructure can introduce extrinsic defects, altering the band alignment, introducing trapping states, and potentially creating semiconductor–metal transitions, which would significantly degrade its desired electronic and optical properties like carrier injection, photocurrent generation, and bandgap energy, ultimately reducing its performance in optoelectronic devices. Defects, specifically metal-like defects, can drastically decrease the Schottky barrier height in MoS_2_/metal contacts, leading to strong Fermi level pinning [31]. This is a direct parallel to how unreacted tellurium (an impurity) could introduce similar defects at the MoS_2_/PtTe_2_ interface, creating undesirable states that pin the Fermi level and disrupt the ideal band alignment. The combined effects of reduced charge carrier mobility, altered band structure, and increased charge carrier recombination due to unreacted Te would lead to significantly degraded electronic and optical performance [32]. The functional performance of a heterostructure is entirely dependent on its band alignment. A study on MoS_2_/PtSe_2_ heterojunctions confirms that these structures have a Type-I band alignment, which is essential for effective charge separation [33]. Unreacted tellurium at the interface would disrupt this ideal alignment by creating additional energy levels or a disordered region that acts as a recombination pathway, rather than a charge separation layer. This would severely impact the device’s ability to efficiently separate electron–hole pairs generated by light, a fundamental requirement for photodetectors and solar cells.

The surface chemistry of MoS_2_ grown by CVD was also analyzed by XPS and used to measure the binding energies of Mo and S. The Mo 3d and S 2s core level peak regions of the as-grown MoS_2_ are shown in Figure 6e,f. The Mo 3d shows three peaks at 228.8 eV, 232.1 eV, and 235.5 eV. The first two peaks are attributed to the doublet Mo 3d5/2 and Mo 3d3/2, respectively, correlating to the Mo^4+^ state in MoS_2_. The third peak of the Mo 3d core level peak at 235.5 eV is attributed to the Mo^6+^ state of MoO_3_. The fitted curves of the doublet Mo^6+^ 3d3/2 and Mo^6+^ 3d5/2 are indicated by the blue line in Figure 6e. This result implies the existence of the Mo oxide state in the MoS_2_ grown on sapphire. There are two possibilities for the existence of MoO_3_. The first one might be related to sulfur deficiency (S vacancy) during CVD growth [28]. After exposing the grown MoS_2_ film to air, the sulfur vacancy will react easily with oxygen to form MoO_3_. The second possibility may be the Mo termination with oxygen of Al–O (sapphire substrate) during MoS_2_ growth. The S 2p spectrum (Figure 6f) has been resolved into two peaks at 163.5 eV and 162.2 eV for S 2p1/2 and 2p3/2, respectively. The presence of a Mo^6+^ state, indicative of MoO_3_, significantly impacts the performance of MoS_2_ devices by degrading carrier mobility and altering the band alignment. This oxidation, whether from sulfur vacancies or oxygen from the substrate, introduces defects that create new energy levels within the MoS_2_ bandgap, acting as charge traps. The concept of defects reducing carrier mobility through scattering is a fundamental principle of condensed matter physics. Specific to MoS_2_, this effect has been quantified. A quantitative increase in defect density directly reflects a decrease in carrier mobility, providing a strong foundation for the explanation that MoO_3_ defects act as scattering centers [34]. MoO_3_, as a new phase, creates a heterojunction with the pristine MoS_2_. The presence of the two phases fundamentally alters the electronic structure, leading to a different band alignment, which would be a major performance issue for applications if not controlled [35].

### 3.5. Optical Properties (Transmittance and Absorbance)

**Figure 7:**
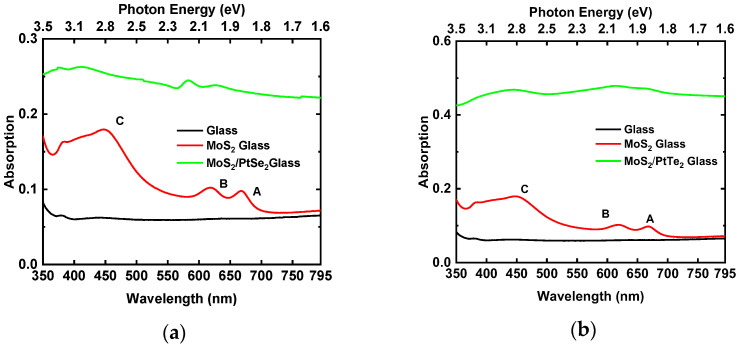
Absorbance spectra of (**a**) MoS_2_ and MoS_2_/PtSe_2_ heterostructure, and (**b**) MoS_2_ and MoS_2_/PtTe_2_ heterostructure.

The absorbance spectra of MoS_2_ and MoS_2_/PtSe_2_ and MoS_2_/PtTe_2_ heterostructures are shown in Figure 7a,b, respectively. Red lines show typical A, B, and C exciton resonance peaks of MoS_2_ at 1.85 eV, 2.00 eV, and 2.78 eV. The MoS_2_ sample is transparent below 1.65 eV. The optical absorbance of MoS_2_ flakes has two prominent narrow peaks occurring at wavelengths ∼605 nm and ∼660 nm that correspond to the absorption due to the direct transitions at the K point of the Brillouin zone, associated with the generation of the B and A excitons, respectively [22]. While the position of the A exciton peak wavelength monotonically red-shifts with the number of layers, the B exciton peak wavelength remains almost unaltered [29]. The spectra also show a broad peak around 440 nm. This feature is typically not observed in photoluminescence experiments, which mostly use an excitation wavelength of ∼500 nm. Recent reflectance and photocurrent spectroscopy experiments, however, present this feature (referred to as a C-exciton peak), whose origin is still a subject of debate [23,24]. The position of this feature strongly depends on the number of layers.

PtSe_2_ and PtTe_2_ have a broad absorption band at 1.0–3.1 eV with no discernible exciton peaks. The spectrum of the fabricated heterostructure shows absorption features from both materials. In the MoS_2_/PtSe_2_ heterostructure, there appears to be a blue-shift in the A and B excitons to ~631 nm and ~584 nm, respectively, while the C exciton broadens. As for the MoS_2_/PtTe_2_ heterostructure, this broadening effect is heightened, and we can no longer observe the well-defined exciton peaks of MoS_2_.

## 4. Conclusions

Vertical heterostructures MoS_2_/PtSe_2_ and MoS_2_/PtTe_2_ were successfully created. The heterostructures were assembled using a method that combines chemical vapor deposition (CVD) for nanolayers/flakes, mechanical exfoliation, and dry transfer. The successful creation of the MoS_2_/PtSe_2_ and MoS_2_/PtTe_2_ heterostructures was confirmed through analyses from Raman spectroscopy, XPS, optical spectrophotometry, and fluorescence spectroscopy. The presence of strong interlayer interactions within the MoS_2_/PtSe_2_ and MoS_2_/PtTe_2_ heterostructures was confirmed based on photoluminescence (PL) measurements.

## Data Availability

The data presented in this study are available on request from the corresponding author.

## Abbreviations

The following abbreviations are used in this manuscript:

2D	Two-dimensional
AFM	Atomic force microscopy
FWHM	Full width at half-maximum
MoS_2_	Molybdenum disulfide
PL	Photoluminescence
PtSe_2_	Platinum diselenide
PtTe_2_	Platinum ditelluride
TMDCs	Transition metal dichalcogenides
TRT	Thermal release tape
XPS	X-ray photoelectron spectroscopy
